# Development of a screening score for Hemophagocytic Lymphohistiocytosis among pediatric patients with acute infection of Epstein-Barr virus

**DOI:** 10.3389/fimmu.2022.981251

**Published:** 2022-09-12

**Authors:** Xun Li, Haipeng Yan, Zhenghui Xiao, Ting Luo, Longlong Xie, Yufan Yang, Ling Gong, Zhexuan Tang, Jiaotian Huang, Xinping Zhang, Mincui Zheng, Zhenya Yao, Ping Zang, Desheng Zhu, Xiulan Lu

**Affiliations:** ^1^ Pediatrics Research Institute of Hunan Province and Department of Pediatric Intensive Care Unit (PICU), Hunan Children’s Hospital, Changsha, China; ^2^ Hunan Provincial Key Laboratory of Emergency Medicine for Children, Hunan Children's Hospital, Changsha, China; ^3^ Department of Pediatric Intensive Care Unit (PICU), Hunan Children’s Hospital, Changsha, China; ^4^ Department of Pediatric Hematology, Hunan Children’s Hospital, Changsha, China

**Keywords:** children, diagnose, Epstein-Barr virus, hemophagocytic lymphohistiocytosis, score, screening

## Abstract

**Background and aims:**

Deciding when to suspect hemophagocytic lymphohistiocytosis (HLH) and perform diagnostic tests in patients with acute infection of Epstein-Barr virus (EBV) is challenging, given the high prevalence of EBV infection, the life-threatening risk of EBV-HLH, the relatively low incidence of EBV-HLH, and the wide spectrum of disease presentations. The aim of this study was to develop an EBV-HLH screening model for pediatric patients diagnosed with acute infection of EBV.

**Methods:**

An inpatient cohort with 3183 pediatric patients who were diagnosed with active infection of EBV was used to construct and validate the EBV-HLH screening score model. The model parameters were selected from common laboratory parameters using the method of Akaike Information Criterion-optimal selection through cross-validation under logistic regression. Performance of the score was evaluated and compared with the performance of screening methods using the number of cytopenias lineages.

**Results:**

The EBV-HLH screening score has five parameters, including hemoglobin, platelet, neutrophil, albumin, and lactate dehydrogenase. Using a cut-of value of 29, the scoring model had a sensitivity of 89.2% and a specificity of 89.5% in the validation set. The false negative rate, false positive rate, positive predictive value, and negative predictive value in the validation set was 10.8%, 10.5%, 26.8%, and 99.5%, respectively, similar to that of the training set.

**Conclusions:**

With five common laboratory parameters, the EBV-HLH score provides a simple tool to assist the identification of EBV patients who require further evaluation of HLH. Further studies are needed to evaluate the generalizability of the score and optimize the diagnose process for EBV-HLH.

## Background

Epstein-Barr virus (EBV) establishes persistent infection in almost 90% of the people worldwide, mostly asymptomatic (1, 2). However, once developed into EBV-related hemophagocytic lymphohistiocytosis (EBV-HLH), it becomes life-threatening. HLH comprises a heterogeneous class of disorders characterized by extreme immune activation (3). HLH can be classified as primary (genetic) or secondary (acquired), and EBV infection was found to be implicated in the onset of both primary and secondary HLH (4). EBV-HLH is one of the most common type of HLH in childhood, especially in Asia area (4, 5). One multi-state clinical trial included 369 pediatric patients with HLH, and found that EBV infection was associated with 20% (n=74) of the patients (6). One study from United States found that approximately 1/3 of the HLH patients were positive for EBV (7). In one Chinese series, EBV infection was found in 94.7% (90/95) of the pediatric HLH patients (8). One study from Vietnam found that 47% (40/85) of the pediatric HLH patients were EBV positive (9). Like all types of HLH, EBV-HLH can rapidly deteriorate and lead to multiple organ failure or even death. Timely diagnosis and early administration of effective therapy are essential for survival (10–12).

The consequence of EBV-HLH could be life-threatening, whilst the diagnose of EBV-HLH is challenging. The diagnose of HLH can be established if a molecular (genetic) diagnosis consistent with HLH or five out of eight criteria fulfilled, which include fever, splenomegaly, cytopenias affecting 2 or 3 lineages, hypertriglyceridemia and/or hypofibrinogenemia, hemophagocytosis, low or absent NK-cell activity, hyperferritinemia, and elevated soluble CD25 (3). Some of these criteria, like genetic test, hemophagocytosis, ferritin, NK-cell activity, and soluble CD25, were unlikely to be tested unless HLH or other relevant diseases were suspected. Our previous study showed that the performance of adequate diagnostic tests for HLH is the most important contribute factor for early diagnosis of HLH (13). However, deciding when to suspect HLH and perform diagnostic tests in patients with EBV is challenging, given the high prevalence of EBV infection, the relatively low incidence of EBV-HLH, and the wide spectrum of disease presentations. It has been suggested that HLH should be suspected when any of the diagnostic, typical, and unique features of HLH are present (5, 12, 14). For example, cytopenias of more than 2 lineages may raise a suspicion for HLH. However, since the diagnostic and typical features of HLH may nonspecific and could been seen in patients with other disorders, the efficacy of suspecting HLH when any of the HLH feature is presented needs further evaluation. Moreover, in order to improve the diagnostic process of EBV-HLH, it is imperative to develop evidence based methods for the identification of high risk patients who would be benefit from the HLH-diagnostic test. In this study, we proposed that developing a screening tool for EBV-HLH would help clinical decision on when to suspect HLH among children with acute EBV infection.

The aim of this study was to develop an EBV-HLH screening model for pediatric patients diagnosed with acute infection of EBV. The strategy of our model development had two considerations: first, to be easily used in clinical practice, the model parameters should be selected from the most prevalently checked laboratory tests; second, for the ease of use, the model should be transformed into a scoring criteria. We extracted clinical data of pediatric patients diagnosed with acute infection of EBV from Hunan Children’s Hospital; the outcome of interest was the diagnose of HLH during hospitalization; laboratory tests that had been check on more than 90% patients with acute infection of EBV were selected as candidate model parameters; a logistic model were developed based on the method of Akaike Information Criterion (AIC)-optimal selection through cross-validation (15), and then this primary model was transformed into a scoring criteria, with a suggested cut-off point. The model was developed in a training set (70% of study population), and was validated in a validation set (30% of the study population). The performance of the screening score model was compared with the performance of the screening method based on the number of cytopenias lineages. The EBV-HLH scoring criteria could serve as a simple tool for the screening of HLH among pediatric patients with acute infection of EBV.

## Methods

### Study population

Pediatric inpatients from Hunan Children’s Hospital diagnosed with acute infection of EBV between January 2017 and December 2021 were included in this study. The disease to be screened was the diagnose of EBV-HLH during hospitalization. Patients with undetermined EBV infection status (cannot distinguish past infection or acute infection) and patients with undetermined EBV-HLH were excluded. Acute infection of EBV were diagnosed by EBV DNA copy number (EBV DNA>1000 copies/ml) or serological profiles (VCA-IgM positive and VCA-IgG positive) (16). HLH were diagnosed according to the HLH-2004 diagnostic criteria (3).

The study protocol was reviewed and approved by the Medical Ethics Committee of the Hunan Children’s Hospital (HCHLL-2019-40 and HCHLL-2022-50) and have been performed in accordance with the ethical standards as laid down in the 1964 Declaration of Helsinki and its later amendments or comparable ethical standards. The requirement for written informed consent was waived by the Medical Ethics Committee of the Hunan Children’s Hospital.

### Variables and data collection

This study investigated variables from the routing blood test, liver and kidney function tests, and cardiac enzymes tests as candidate screening parameters, because these three types of blood tests were conducted in most of the inpatients in the study site. A total of 37 lab parameters were examined, including white blood cell count, lymphocyte ratio, neutrophil ratio, monocyte ratio, eosinophil ratio, basophil ratio, mean red blood cell volume, hematocrit, mean RBC hemoglobin concentration, platelet count, hemoglobin, red blood cell count, mean RBC hemoglobin, neutrophil count, monocyte count, lymphocyte count, eosinophil count, basophil count, total protein, albumin, total bilirubin, direct bilirubin, indirect bilirubin, aspartate aminotransferase (AST), alanine aminotransferase (ALT), total bile acids, AST/ALT, albumin/globulin, globulin, lactate dehydrogenase (LDH),creatinine (CREA), uric acid, blood urea nitrogen (BUN), BUN/CREA, myoglobin, creatine kinase (CK), and CK-MB. These blood tests results were available from more than 90% of hospitalized EBV patients in the study site. For each patients, the first test result for each laboratory parameter were extracted from electronic medical record and was used for the development of screening models.

### Statistical analysis

Data were presented as absolute values and percentages, or mean (standard deviation, SD), or quartiles (median, Q1, and Q3), as appropriate. Between-group comparisons were conducted using chi-squared test or *t*-test, as appropriate. All tests were set two-tailed with a type 1 error rate fixed at 5%. Missing data was not imputed. Statistical analyses were performed using SAS 9.4 (SAS Institute, Inc.,Cary, NC) and R 4.1.3.

To visualize clusters between patients and to identify the most prominent drivers of difference between EBV-HLH and EBV-nonHLH patients, two clustering methods with dimension reduction, namely the principal component analysis (PCA, unsupervised clustering) and the partial lest squares discriminant analysis (PLS-DA, supervised clustering) were conducted. The “mixOmics” package in R 4.1.3 was used to perform the PCA and PLS-DA (17).

The study cohort was randomly partitioned into a training (70%) and validation (30%) set by using the Proc surveyselect procedure in SAS 9.4 software. Logistic regression was used to build the screening score model. We first developed a model based on continuous variables (Model 1). For the ease of use, the continuous variables in Model 1 were categorized into binary variables and a second model (Model 2) was developed using binary predictors; finally, a scoring model (Model 3) was developed based on Model 2.

In Model 1, variables were selected applying the method of Akaike Information Criterion (AIC)-optimal selection through cross-validation (15). The AIC-optimal stepwise selection utilizes AIC as the criterion for variable importance. The training set was disjoint into 10 subsets, and the cross-validation was repeated by 3 times. In each iteration, a lists of influential variables was selected by stepwise logistic regression. Variables were then ranked by their frequency appearing in the AIC-optimal lists obtained from cross-validation iterations. In the next step, models were built by sequentially adding the variables with the same frequency, and the model showed an optimal averaged area under the receiver operating characteristic curve (AUC) was selected as the candidate model. As hemoglobin, platelet, and neutrophil are items in the diagnostic criteria of HLH, and they are most frequently tested laboratory parameters, we also investigated their performance in the screening model by adding them to the model if they had not been selected in the AIC-optimal selection through cross-validation. If the AUC and AIC were improved after adding hemoglobin, platelet, and neutrophil as model variables, these variables would be kept in the preliminary model (Model 1).

Continues variables from Model 1 were then categorized into binary variables. For hemoglobin, platelet, and neutrophil, the criteria from the HLH-2004 criteria was used as cut-off values; for other variables, the cut-off point was determined by the maximum value of Youden’s index (the sum of sensitivity and specificity minus one) from the receiver operating characteristic (ROC) analysis. The second screening model (Model 2) was developed by logistic regression using binary variables as predictors.

The coefficients resulting from the Model 2 were used to assign score points for the construction of a scoring model, by multiplying the coefficient (beta value) for each variable by 10 and round off to the nearest integer (18). The sum of scores of each variable was calculated for each individual in the training set, and a third logistic model (Model 3) was developed using the score as the single prediction variable. The cut-off point of the score was determined by the maximum value of Youden’s index, and the model performance of applying this cut-off point was evaluated.

As cytopenias of more than 2 lineages or of 3 lineages were clinically used as clues for suspecting HLH. We also investigated the performance of using cytopenias of ≥2 lineages and cytopenias of 3 lineages as screening tests (Model 4 and Model 5), and compared them with that of the scoring model developed in this study.

AUC was used to evaluate discrimination of logistic regression models. To assess the model performance, the accuracy, sensitivity, specificity, false negative rate (FNR), false positive rate (FPR), positive predictive value (PPV), and negative predictive value (NPV) were calculated.

### Sensitivity analysis

According to the inclusion criteria of this study, acute infection of EBV were diagnosed by the copy number of EBV-DNA or by a serological test. We categorized the study population into two sub-populations according to the types of EBV diagnostic test and conducted a sensitivity analysis to assess the performance of the scoring model in the sub-populations.

## Results

During the study period, 3523 pediatric inpatients were tested positive for active EBV infection. Among these, 8 patients were excluded because they were clinically suspected for EBV-HLH but the diagnoses were not confirmed; 332 patients were excluded because missing essential laboratory data. A total of 3183 pediatric EBV-positive pediatric patients were included (**Supplementary Figure S1**). **Table 1** summarized the general characteristics of the study population. The incidence of EBV-HLH in the inpatient EBV cohort were 4.62%. The distributions of age, sex, and incidence of EBV-HLH in the training and validation cohort were not statistically significant from each other (*P*>0.05, **Table 1**).

**Table 1:** Summary statistics of the study population.

	Total	Training set	Validation set	*P*
N	3183	2229	954	
Age, mean (SD)	4.27 (3.06)	4.26 (3.04)	4.27 (3.08)	0.8739
Sex, n (%)				
Female	1289 (40.5)	904 (40.6)	385 (40.4)	0.9161
Male	1894 (59.5)	1325 (59.4)	569 (59.6)	
EBV-HLH, n (%)				
Yes	147 (4.62)	99 (4.4)	48 (5.0)	0.4675
No	3036 (95.38)	2130 (95.6)	906 (95.0)	

**Figure 1:**
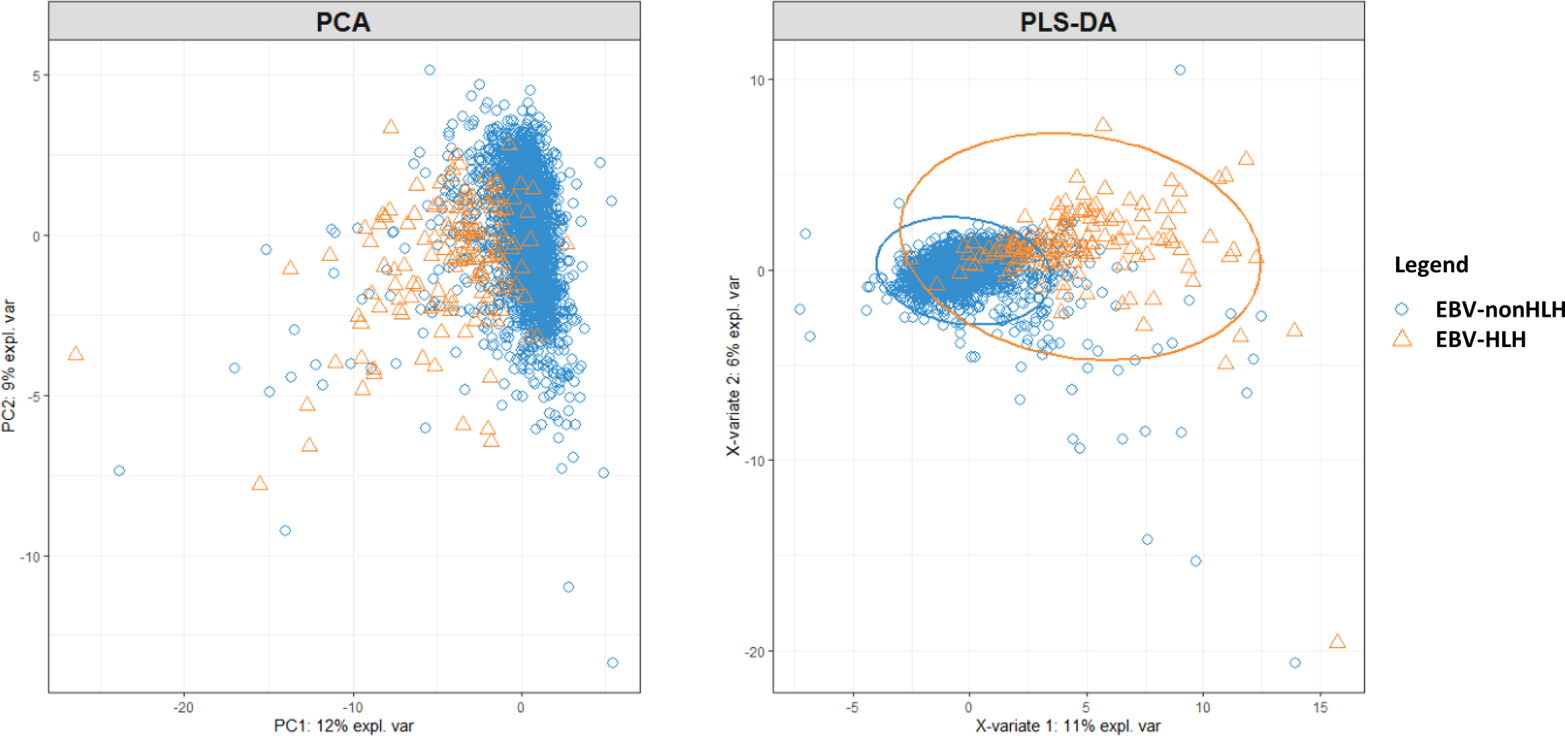
PCA and PLS-DA on 37 laboratory parameters from pediatric patients with acute EBV infection with and without HLH.

Among the 37 investigated laboratory parameters, 32 showed to be significantly different between EBV-HLH and EBV-nonHLH patients (*P <*0.05, **Supplementary Table S1**). The PCA and PLS-DA plots were presented in **Figure 1**. Total bilirubin, direct bilirubin, and indirect bilirubin were the top three defining parameters in the PCA; and LDH, hematocrit, and hemoglobin were the top three defining parameters in the PLS-DA. Both PCA and PLS-DA showed limited ability to separating the EBV-HLH patients from EBV patients (**Figure 1**).

**Figure 2:**
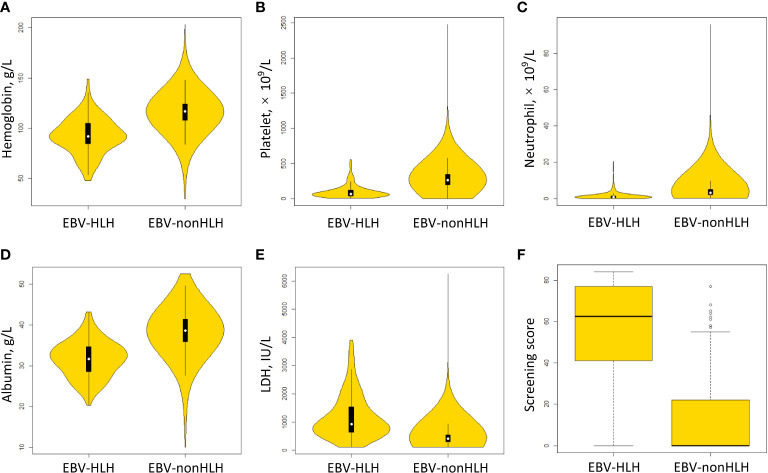
Distribution of scoring parameters and the screening score in EBV patients with and without HLH. **(A)** hemoglobin. **(B)** platelet. **(C)** neutrophil. **(D)** albumin. **(E)** LDH, lactate dehydrogenase. **(F)** screening score.

The model build from the method of AIC-optimal selection through cross-validation included three variables, including platelet, albumin, and LDH. After adding hemoglobin and neutrophil to the model, the AIC was improved from 445.6 to 392.5, and the AUC was improved from 93.1% to 95.2%. Therefore a preliminary model (Model 1) with five parameters, namely hemoglobin, platelet, neutrophil, albumin, and LDH, were determined. The model parameters were presented in **Supplementary Table S2**. **Figures 2A–E** showed the distribution of the selected parameters in EBV patients with and without HLH.

**Figure 3:**
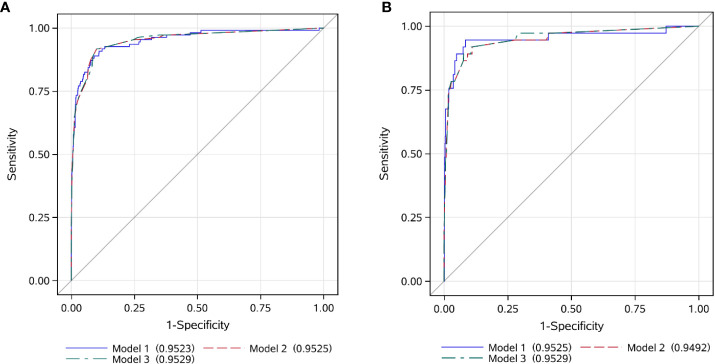
ROC curves of the EBV-HLH screening score in the training and validation set. **(A)** training set; **(B)** validation set.

Following the method described above, Model 2 was developed using categorized variables and Model 3 was developed using a score as the predictive variable. The AUCs of three models were similar between the training and validation sets (range from 94.9% to 95.3%) (**Figure 3**). The median screening score in EBV-nonHLH patients was 0 (Q1, Q3: 0, 22), and the median score in EBV-HLH patients was 62.5 (Q1, Q3: 41, 77; *P*<0.0001) (**Figure 2F**). Using a cut-of value of 29, the scoring system had a sensitivity of 91.7% and a specificity of 90.0% in the training set, and a sensitivity of 89.2% and a specificity of 89.5% in the validation set. The FNR, FPR, PPV, and NPV in the validation set was 10.8%, 10.5%, 26.8%, and 99.5%, respectively, similar to that of the training set (**Table 2**). The screening methods using the number of cytopenias lineages (≥ 2 or =3) showed high specificities (>97%), however, with low sensitivities (57.8% for cytopenias of ≥2 lineages and 25.7% for cytopenias of 3 lineages, training set), demonstrating the superiority of the newly developed scoring model. The scoring criteria of the screening score was presented in **Table 3**.

**Table 2:** Performance of five models for the screening of EBV-HLH among pediatric patients positive for EBV test.

Model	Data set	AUC(%)	Accuracy (%)	Sensitivity(%)	Specificity(%)	FNR(%)	FPR(%)	PPV(%)	NPV(%)	Youden
Model 1^a^: 5 continuous parameters	Training	95.2	89.1	90.8	89.0	9.2	11	31.2	99.4	0.80
	Validation	95.3	88.4	94.6	88.1	5.4	11.9	25.5	99.7	0.83
Model 2^b^: 5 categorical parameters	Training	95.2	90.1	91.7	90.0	8.3	10	33.4	99.5	0.82
	Validation	94.9	89.5	89.2	89.5	10.8	10.5	26.8	99.5	0.79
Model 3^c^: scoring model based on 5 categorical parameters	Training	95.3	90.1	91.7	90.0	8.3	10	33.4	99.5	0.82
	Validation	95.3	89.5	89.2	89.5	10.8	10.5	26.8	99.5	0.79
Model 4^d^: Cytopenias of ≥2 lineages	Training	–	95.5	57.8	97.6	42.2	2.4	56.8	97.7	–
	Validation	–	95.8	59.5	97.3	40.5	2.7	48.9	98.2	–
Model 5^e^: Cytopenias of 3 lineages	Training	–	95.6	25.7	99.4	74.3	0.6	71.8	96.1	–
	Validation	–	96.3	27.0	99.3	73	0.7	62.5	96.9	–

AUC, area under the curve; DI, diagnostic index; EBV, Epstein-Barr virus; HLH, hemophagocytic lymphohistiocytosis; FNR, false negative rate; FPR, false positive rate; NPV, negative predictive value; PPV, positive predictive value; Youden, Youden’s index.

^a^Model 1: logit P = 9.32 - 0.04 × hemoglobin - 0.008 × platelet- 0.31 × neutrophil - 0.195 × albumin +0.002 × lactate dehydrogenase; The optimal cut-off probability = 0.0447, which means that if the predicted probability ≥ 0.0447, that patients is identified as high risk for EBV-HLH.

^b^Model 2: logit P = -6.159 + 0.910 × (hemoglobin < 90 g/L) + 1.705 × (platelet < 100×10^9^/L)+ 2.108 × (neutrophil < 1.0×10^9^/L) + 2.22 × (albumin < 38.5 g/L) + 2.043 × (lactate dehydrogenase > 637 IU/L); If the parameter value meet the criteria in the brackets then assign the value of 1, else 0. The optimal cut-off probability = 0.0461.

^c^Model 3: logit P = -6.16 + 0.1× score; The scoring criteria was presented in **Table 3**. The optimal cut-off probability = 0.042.

^d^Model 4: if cytopenias of ≥ 2 lineage, then the individual was identified as high risk for EBV-HLH.

^e^Model 5: if cytopenias of 3 lineage, then the individual was identified as high risk for EBV-HLH.

**Table 3:** EBV-HLH screening score.

Parameter	Criteria	Score assigned
Hemoglobin, g/L	≥ 90	0
	< 90	7
Platelet, ×10^9^/L	≥ 100	0
	< 100	20
Neutrophil, ×10^9^/L	≥ 1.0	0
	< 1.0	16
Albumin, g/L	≥ 38.5	0
	< 38.5	22
Lactate dehydrogenase, IU/L	≤ 637	0
	> 637	19

The identified best cut-off value for screening score was 29, corresponding to a sensitivity of 91.7%, a specificity of 90.0% in the training set, and a sensitivity of 89.2%, and a specificity of 89.5% in the validation set.

Among the 3183 patients included in this study, 2989 had available lab data to calculate the screening score. Among these patients, 2028 were diagnosed with acute infection of EBV according to EBV-DNA copy numbers, and 961 were diagnosed by serological profiles. The performance of the scoring model in the sub-populations divided by two types of EBV tests were presented in **Supplementary Table S3**. Although the model accuracy and specificity among patients diagnosed by serology tests (accuracy=94.2%, specificity=94.2%) were higher than that among patients diagnosed by EBV DNA copy numbers (accuracy=87.9%, specificity=87.6%), the model showed better sensitivity in the EBV DNA-diagnosed subpopulation (91.4%) than that in the serology-diagnosed subpopulation (85.7%).

## Discussion

In this study, we developed a score for the screening of HLH among pediatric patients with acute EBV infection. The score model has five parameters, including hemoglobin, platelet, neutrophil, albumin, and LDH. The sensitivity and specificity of the score model in the validation set were 89.2% and 89.5%, respectively. The performance of the screening score was stable in the training and validation set, as well as in the two sub-populations that using different EBV diagnostic tests.

Patients with acute infection of EBV may develop some of the features of HLH, like fever, splenomegaly, and cytopenias (5). Therefore, HLH may be difficult to distinguish from EBV. In clinical practice, patients who presents more sever manifestations of disease may undergo further evaluation for suspected HLH. However, the diagnosis of HLH could be delayed because the diagnosis of HLH requires multiple tests and there are no clinical thresholds that guide the routine practice for the identification of patients who need further evaluations for possible HLH. To improve the diagnostic procedures of HLH, researchers had been working on the development of new diagnostic tools. Smits et al. (2021) identified a minimal parameter set for the prediction of HLH (19); the minimal parameter set consisting phagocytosis, splenomegaly, cytopenias, increased ferritin, and increased triglycerides/low fibrinogen, with a sensitivity of 95% and a specificity of 94%. One of the strengths of the minimal parameter set is it excludes the checking of NK activity and soluble CD25, which cannot be measured in routine practice in many settings and can be time consuming. The HScore was developed for the diagnosis of reactive HLH in adult (18), and has been validated in several adult and pediatric populations (20, 21). HScore has 9 variables, including known underlying immunosuppression, high temperature, organomegaly, triglyceride, ferritin, serum glutamic oxaloacetic transaminase, fibrinogen levels, cytopenia, hemophagocytosis features on bone marrow aspirate. HScore also excludes the use of NK activity and soluble CD25. Although the utility and generalizability of the minimal parameter set and the HScore need further investigation and validation, the promising results suggested that alternative diagnostic tools for HLH may be developed in the near future. However, these two criteria still consist a variety of parameters, including clinical, biologic, and cytologic variables. The question of when to suspect HLH and take the diagnostic tests remained.

This study used a data-driven approach to develop the screen model for EBV-HLH. Candidate model parameters were selected from the most frequently checked laboratory parameters. The purpose of this approach was that the screening results could be obtained during routine lab tests, which would assist in the first step of identifying suspected HLH among pediatric patients with EBV. The screening score included five parameters, including three lineages of cytopenias, albumin, and LDH. Cytopenias in HLH can be explained by direct hemophagocytosis, active disseminated intravascular coagulation, and high concentrations of tumor necrosis factor-α and interferon–γ, which stimulated the process of consumptive micropinocytosis (22, 23). Decreased albumin and elevated LDH can attributed to the organ dysfunction in HLH. Cytopenias were also parameters from the HLH-2004 diagnostic criteria, and this feature was developed in up to 100% of patients with HLH (13). Since cytopenias were typical features of HLH, we investigated the performance of ≥2 or 3 lineages of cytopenias as screening tests for EBV-HLH. Our results showed that these screening methods had high specificities (up to 99.4%), however, with low sensitivities (range from 25.7% to 59.5%), suggesting cytopenias along were not satisfactory screening tools for HLH among patients with EBV. Decreased albumin and elevated LDH had been observed in both pediatric and adult HLH (24, 25), and was found to be associated with poor prognosis (25–27). In a pediatric HLH case series, 113 of 116 patients (97.4%) had decreased albumin, and 110 of 111 patients (99.1%) had elevated LDH (26). In our study, the identified cut-off value for albumin was 38.5.g/L; and 23.7% of the patients with EBV-nonHLH and 86.3% of the patients with EBV-HLH had albumin < 38.5.g/L in their first albumin test (*P*<0.05). The cut-off value for albumin was similar to the normal reference range in the local clinical laboratory (<35 g/L). For LDH, the cut-off value in the screening score (>637 IU/L) was higher than the normal reference range in the local clinical laboratory (>450 IU/L). In the study population, 44.43% of patients with acute EBV infection had LDH > 450; 15.09% of patients with EBV-nonHLH had LDH >637 IU/L, and 76.03% of patients with EBV-HLH had LDH >637 IU/L (*P*<0.05). In other words, elevated LDH was developed in nearly half of the EBV patients, but those with HLH had more sever manifestation, which leads to the higher thresholds of screening score than the local lab reference range.

Strengths of this study included it used a data-driven approach to select model parameters, which could minimize subjective bias. Dozens of variables had been found to be associated with HLH, many of them were proposed to be used in the diagnosis of HLH; to build a robust model and avoid overfitting, we used the method of AIC-optimal selection through cross-validation, which was developed to overcome the drawbacks of stepwise selection and selection over a single sample (15). The second strength was the simplicity of the screening score. With only five parameters which are frequently checked in routine practice, the score is easy to use; it could be embedded into the electronic medical record system and provide early warning for EBV-HLH.

This study has several limitations. First, it was a single center study, which could subject to selection bias. The screening score need to be validated in other populations, and the cut-off point might need to be adjusted to different populations. Second, this study only included inpatients, which also could subject to selection bias. HLH is a severe syndrome which in most cases requires hospitalization, therefore, we only included inpatients. Besides, patients who diagnosed with acute infection with EBV in the outpatient department were not followed up for the development of HLH. Since the diagnostic outcome could not be confirmed in outpatients, data from this population were excluded from this study. Therefore, according to the design of this study, the EBV-HLH score was supposed to be used in pediatric inpatients. Further studies are needed to investigate whether the score could be used to identify patients with early EBV-HLH before the need of hospitalization. Third, this study excluded patients with suspected but unconfirmed HLH and patients whose infection status of EBV could not be determined. Although the excluded population was relatively small and should had limited influence on the performance of the score, further validation studies are essential for the evaluation of the performance and generalizability of the EBV-HLH screening score. Fourth, the EBV-HLH screening score was supposed to be used in patients diagnosed with acute infection of EBV, even though the diagnose of EBV infection could be delayed. Although suspecting for EBV-HLH after the diagnosis of EBV infection was reasonable in clinical practice, it could delay the diagnosis of HLH. To promote early identification of EBV-HLH, further studies are needed to optimize the screening procedure and targeted screening population.

## Conclusion

In conclusion, we developed a screening score for the identification of HLH among pediatric patients with acute infection of EBV. With five common laboratory parameters, the EBV-HLH score provides a simple tool to assist the identification of EBV patients who require further evaluation of HLH. Further studies are needed to evaluate the generalizability of the score and optimize the diagnose process for EBV-HLH.

## Data availability statement

The raw data supporting the conclusions of this article will be made available by the authors, without undue reservation.

## Ethics statement

The studies involving human participants were reviewed and approved by The Medical Ethics Committee of the Hunan Children’s Hospital. Written informed consent from the participants’ legal guardian/next of kin was not required to participate in this study in accordance with the national legislation and the institutional requirements.

## Author contributions

XLi, HY, and ZX contributed to the study’s conception, analysed and interpreted the data, and wrote the manuscript. TL, LX, YY, LG, ZT, JH, XZ, and MZ contributed to the study design, interpreted the data, and revised the manuscript. ZY, PZ, and DZ performed chart reviews, interpreted the data, and revised the manuscript. XLu designed the study, interpreted the data, and revised the manuscript. All authors read and approved the final manuscript. The corresponding author (XLu) had full access to all the data in the study and takes responsibility for the integrity of the data and the accuracy of the data analysis. All authors approved the final version of the manuscript.

## Funding

This study was supported by the Hunan Provincial Science and Technology Department Project (2020SK1014-3 and 2020SK2114, grant to XLu; 2018SK2135, grant to ZX), the Hunan Provincial Key Laboratory of Emergency Medicine for Children (No. 2018TP1028, grant to ZX), the National Natural Science Foundation of China (Young Scientists Fund, No. 82102285, grant to XLi), the Natural Science Foundation of Hunan Province (No. 2021JJ40270, grant to XLi), and the Scientific Research Project of Hunan Provincial Health Commission (No. 202112050360, grant to XLi). The study sponsors have no role in the study design, data collection, data analysis, data interpretation, or writing of the report.

## Acknowledgments

The authors would like to thank Dan Yang and Yu Tian for their assistance in data management.

## Conflict of interest

The authors declare that the research was conducted in the absence of any commercial or financial relationships that could be construed as a potential conflict of interest.

## Publisher’s note

All claims expressed in this article are solely those of the authors and do not necessarily represent those of their affiliated organizations, or those of the publisher, the editors and the reviewers. Any product that may be evaluated in this article, or claim that may be made by its manufacturer, is not guaranteed or endorsed by the publisher.
