# Supplementary material for: Development of a screening score for Hemophagocytic Lymphohistiocytosis among pediatric patients with acute infection of Epstein-Barr virus
Source: Front Immunol. 2022 Sep 12;13:981251. doi: 10.3389/fimmu.2022.981251 (PMC9510983; doi:10.3389/fimmu.2022.981251)
Supplement: Supplementary file 1 [file DataSheet_1.docx]

**Additional file 1**

**Supplemental Figure S1.** Study flow chart

**Supplemental Table S1.** Distribution of investigated laboratory parameters among pediatric patients positive for EBV according to HLH status

**Supplemental Table S2.** Estimated model parameters in the selected multivariable Logistic regression models

**Supplemental Table S3.** Performance of the screening score model in sub-population defined by different EBV diagnostic tests

**EBV-positive**

N=3523

**Included**

n=3183

Excluded, n=343

1. Cannot confirm HLH, n=8
2. Missing essential laboratory test results, n=332

Training set, n=2229

HLH, n=99

Non-HLH, n=2130

HLH, n=48

Non-HLH, n=906

Validation set, n=954

**Figure S1. Study flow chart**

Table S1 Distribution of investigated laboratory parameters among pediatric patients positive for EBV according to HLH status

| Parameters | EBV-HLH | | |  | EBV-nonHLH | | | *P* |
| --- | --- | --- | --- | --- | --- | --- | --- | --- |
|  | Median | Q1, Q3 | Min, Max |  | Median | Q1, Q3 | Min, Max |  |
| White blood cell count, ×10^9^/L | 2.51 | 1.47, 4.96 | 0.40, 28.94 |  | 9.82 | 6.97, 14.23 | 0.25, 152.20 | <0.0001 |
| Lymphocyte ratio, ×100 | 0.571 | 0.34, 0.70 | 0.02, 0.92 |  | 0.531 | 0.36, 0.65 | 0.03, 0.96 | 0.1171 |
| Neutrophil ratio, ×100 | 0.338 | 0.20, 0.55 | 0.02, 0.91 |  | 0.342 | 0.21, 0.54 | 0.01, 0.95 | 0.5895 |
| Monocyte ratio, ×100 | 0.07 | 0.04, 0.10 | 0.00, 0.21 |  | 0.06 | 0.05, 0.08 | 0.00, 0.32 | 0.0101 |
| Eosinophil ratio, ×100 | 0 | 0.00, 0.00 | 0.00, 0.10 |  | 0.005 | 0.00, 0.02 | 0.00, 0.82 | <0.0001 |
| Basophil ratio, ×100 | 0.002 | 0.00, 0.01 | 0.00, 0.17 |  | 0.003 | 0.00, 0.01 | 0.00, 0.06 | <0.0001 |
| Mean red blood cell volume, fl | 78.4 | 75.30, 81.30 | 53.10, 103.50 |  | 81.9 | 79.00, 84.60 | 50.40, 115.80 | <0.0001 |
| Hematocrit, % | 28.1 | 25.50, 31.50 | 14.60, 46.30 |  | 35.6 | 33.20, 37.80 | 9.50, 58.10 | <0.0001 |
| Mean RBC hemoglobin concentration, g/L | 330 | 323.00, 340.00 | 280.00, 366.00 |  | 327 | 320.00, 334.00 | 246.00, 656.00 | 0.0015 |
| Platelet count, ×10^9^/L | 66 | 36.00, 119.00 | 2.00, 552.00 |  | 262 | 194.00, 345.00 | 1.00, 2475.00 | <0.0001 |
| Hemoglobin, g/L | 92 | 84.00, 105.00 | 48.00, 149.00 |  | 117 | 108.00, 124.00 | 30.00, 203.00 | <0.0001 |
| Red blood cell count, ×10^12^/L | 3.68 | 3.26, 4.06 | 1.41, 5.89 |  | 4.37 | 4.08, 4.67 | 1.08, 7.21 | <0.0001 |
| Mean RBC hemoglobin, pg | 26 | 24.70, 27.20 | 17.70, 34.00 |  | 26.8 | 25.80, 27.80 | 15.40, 56.70 | <0.0001 |
| Neutrophil count, ×10^9^/L | 0.81 | 0.44, 1.45 | 0.02, 20.28 |  | 3.17 | 1.99, 5.11 | 0.03, 95.89 | <0.0001 |
| Monocyte count, ×10^9^/L | 0.15 | 0.08, 0.39 | 0.01, 4.41 |  | 0.56 | 0.37, 0.89 | 0.00, 11.76 | <0.0001 |
| Lymphocyte count, ×10^9^/L | 1.32 | 0.58, 2.96 | 0.04, 14.73 |  | 4.6035 | 2.66, 7.56 | 0.02, 37.27 | <0.0001 |
| Eosinophil count, ×10^9^/L | 0 | 0.00, 0.01 | 0.00, 0.65 |  | 0.05 | 0.01, 0.15 | 0.00, 57.06 | <0.0001 |
| Basophil count, ×10^9^/L | 0.01 | 0.00, 0.02 | 0.00, 0.23 |  | 0.03 | 0.01, 0.05 | 0.00, 2.28 | <0.0001 |
| Total protein, g/L | 55.8 | 49.90, 61.50 | 36.40, 84.00 |  | 66.2 | 62.10, 70.20 | 25.80, 98.00 | <0.0001 |
| Albumin, g/L | 31.7 | 28.50, 34.79 | 20.30, 43.20 |  | 38.7 | 36.00, 41.50 | 10.10, 52.50 | <0.0001 |
| Total bilirubin, μmol/L | 10.7 | 7.28, 50.80 | 1.10, 693.60 |  | 6.65 | 5.10, 9.00 | 1.00, 491.20 | <0.0001 |
| Direct bilirubin, μmol/L | 5.42 | 2.80, 32.30 | 0.50, 640.80 |  | 2.6 | 1.70, 3.70 | 0.00, 243.10 | <0.0001 |
| Indirect bilirubin, μmol/L | 6.1 | 3.70, 12.60 | 0.10, 106.90 |  | 4.1 | 2.90, 5.70 | 0.00, 248.10 | <0.0001 |
| Aspartate aminotransferase (AST), IU/L | 189.7 | 76.00, 411.20 | 10.80, 5765.00 |  | 41 | 27.33, 74.90 | 0.30, 5798.40 | <0.0001 |
| Alanine aminotransferase (ALT), IU/L | 154.2 | 49.10, 314.60 | 3.70, 3368.00 |  | 29.9 | 16.80, 95.80 | 0.74, 5600.00 | <0.0001 |
| Total bile acids, μmol/L | 23.6 | 8.80, 119.10 | 1.10, 242.20 |  | 7.005 | 4.40, 12.00 | 0.10, 308.00 | <0.0001 |
| AST/ALT | 1.43 | 0.93, 2.08 | 0.18, 81.37 |  | 1.23 | 0.78, 1.80 | 0.02, 34.80 | 0.0012 |
| Albumin/Globulin | 1.38 | 1.10, 1.63 | 0.48, 2.35 |  | 1.42 | 1.22, 1.66 | 0.38, 4.28 | 0.0531 |
| Globulin, g/L | 23 | 18.90, 27.90 | 11.80, 51.10 |  | 26.9 | 23.70, 30.80 | 8.30, 58.30 | <0.0001 |
| Lactate dehydrogenase, IU/L | 921.5 | 643.00, 1544.00 | 118.00, 3901.00 |  | 408 | 296.00, 545.00 | 118.00, 6250.00 | <0.0001 |
| Creatinine (CREA), μmol/L | 30 | 23.30, 37.50 | 9.00, 183.00 |  | 29 | 23.60, 35.60 | 4.00, 1359.00 | 0.5220 |
| Uric acid , μmol/L | 208 | 152.00, 277.68 | 14.00, 902.00 |  | 252 | 199.00, 317.00 | 7.00, 934.00 | <0.0001 |
| Urea nitrogen (BUN), μmol/L | 3.53 | 2.78, 4.96 | 1.33, 22.77 |  | 3.34 | 2.58, 4.18 | 0.25, 38.53 | 0.0025 |
| BUN/CREA, ×100 | 0.12 | 0.09, 0.17 | 0.03, 0.29 |  | 0.11 | 0.08, 0.15 | 0.01, 0.88 | 0.0029 |
| Myoglobin, ng/ml | 34.05 | 20.10, 54.80 | 0.00, 310.50 |  | 31.1 | 17.80, 49.30 | 0.00, 1572.00 | 0.2599 |
| Creatine kinase (CK), U/L | 47.5 | 29.90, 94.00 | 7.00, 1015.00 |  | 61 | 42.00, 92.00 | 6.00, 18277.9 | 0.0014 |
| CK-MB, U/L | 17.3 | 11.90, 26.40 | 0.00, 191.20 |  | 13.6 | 9.10, 20.00 | 0.00, 555.70 | <0.0001 |

Table S2. Estimated model parameters in the selected multivariable Logistic regression models

| Parameters | As continuous variables | | |  | As categorical variables | | | |
| --- | --- | --- | --- | --- | --- | --- | --- | --- |
|  | β | SE | *P* |  | Cut-off point | β | SE | *P* |
| Platelet count, 10^9^/L | -0.01 | 0.002 | <0.0001 |  | <100 | 1.7 | 0.312 | <0.0001 |
| Hemoglobin, g/L | -0.04 | 0.008 | <0.0001 |  | <90 | 0.91 | 0.355 | 0.0104 |
| Neutrophil count, 10^9^/L | -0.31 | 0.082 | 0.0001 |  | <1.0 | 2.11 | 0.319 | <0.0001 |
| Albumin, g/L | -0.2 | 0.032 | <0.0001 |  | <35.8 | 2.22 | 0.328 | <0.0001 |
| Lactate dehydrogenase, IU/L | 0 | 0 | <0.0001 |  | >637 | 2.04 | 0.295 | <0.0001 |

Table S3 Performance of the screening score model in sub-population defined by different EBV diagnostic tests

|  | Sub-population | |
| --- | --- | --- |
|  | Diagnosed by EBV DNA copy number  (n=2028) | Diagnosed by serological profiles  (n=961) |
| Accuracy, % | 87.9 | 94.2 |
| Sensitivity, % | 91.4 | 85.7 |
| Specificity, % | 87.6 | 94.2 |
| FNR, % | 8.6 | 14.3 |
| FPR, % | 12.4 | 5.8 |
| PPV, % | 35.2 | 9.8 |
| NPV, % | 99.3 | 99.9 |
